# The association between primary care use and potentially-preventable hospitalization among dual eligibles age 65 and over

**DOI:** 10.1186/s12913-022-08326-2

**Published:** 2022-07-19

**Authors:** N. Loren Oh, Andrew J. Potter, Lindsay M. Sabik, Amal N. Trivedi, Fredric Wolinsky, Brad Wright

**Affiliations:** 1 University of North Carolina at Chapel Hill School of Medicine, Chapel Hill, NC USA; 2 Department of Health Policy and Management, Gillings School of Public Health, University of North Carolina at Chapel Hill, Chapel Hill, USA; 3 Department of Political Science & Criminal Justice, California State University, Chico, USA; 4 Department of Health Policy and Management, Graduate School of Public Health, University of Pittsburgh, Pittsburgh, USA; 5 Department of Health Services, Policy and Practice, School of Public Health, Brown University, Providence, USA; 6 Department of Health Management and Policy, College of Public Health, University of Iowa, Iowa City, USA; 7 Cecil G. Sheps Center for Health Services Research, University of North Carolina at Chapel Hill, 590 Manning Dr. CB 7595, Chapel Hill, NC 27599 USA

**Keywords:** Primary care, Preventable hospitalization, Dual Eligibles, Medicare, Ambulatory care sensitive conditions

## Abstract

**Background:**

Individuals dually-enrolled in Medicare and Medicaid (dual eligibles) are disproportionately sicker, have higher health care costs, and are hospitalized more often for ambulatory care sensitive conditions (ACSCs) than other Medicare beneficiaries. Primary care may reduce ACSC hospitalizations, but this has not been well studied among dual eligibles. We examined the relationship between primary care and ACSC hospitalization among dual eligibles age 65 and older.

**Methods:**

In this observational study, we used 100% Medicare claims data for dual eligibles ages 65 and over from 2012 to 2018 to estimate the likelihood of ACSC hospitalization as a function of primary care visits and other factors. We used linear probability models stratified by rurality, with subgroup analyses for dual eligibles with diabetes or congestive heart failure.

**Results:**

Each additional primary care visit was associated with an 0.05 and 0.09 percentage point decrease in the probability of ACSC hospitalization among urban (95% CI: − 0.059, − 0.044) and rural (95% CI: − 0.10, − 0.08) dual eligibles, respectively. Among dual eligibles with CHF, the relationship was even stronger with decreases of 0.09 percentage points (95% CI: − 0.10, − 0.08) and 0.15 percentage points (95% CI: − 0.17, − 0.13) among urban and rural residents, respectively.

**Conclusions:**

Increased primary care use is associated with lower rates of preventable hospitalizations for dual eligibles age 65 and older, especially for dual eligibles with diabetes and congestive heart failure. In turn, efforts to reduce preventable hospitalizations for this dual-eligible population should consider how to increase access to and use of primary care.

**Supplementary Information:**

The online version contains supplementary material available at 10.1186/s12913-022-08326-2.

## Background

There were 12.2 million individuals dually enrolled in Medicare and Medicaid in 2018 [1]. These dual eligibles include low-income adults over age 65 or with disabilities. Compared to the general Medicare population, they are more likely to have multiple chronic conditions, complex health needs, and poor self-reported health status [2–4]. They are also a high-cost population of interest to policymakers [1, 5]. For example, dual eligibles have above average rates of potentially-preventable hospitalization for ambulatory care sensitive conditions (ACSCs) [6]. From 2007 to 2009, over a quarter of hospitalizations among dual eligibles were for ACSCs, totaling 600,000 potentially-preventable hospitalizations in 2009 with an associated cost of $5.4 billion [7]. Improving care for dual eligibles by bolstering access to primary care may reduce high-cost ACSC hospitalizations.

Access, continuity, comprehensiveness, and care coordination are considered key to providing higher quality, more cost-effective, and more equitable care [8, 9]. Yet, much of US health policy is structured in ways that systematically undervalue primary care and threaten its future [10]. From 2005 to 2015, the per capita supply of primary care physicians in the US decreased, with rural areas experiencing the largest declines [11], while nurse practitioners have increasingly stepped in to meet the growing demand for primary care [12].

A large body of evidence suggests that greater access to primary care is associated with a lower probability of ACSC hospitalization [13–17], but many of these studies used area-level data and may be subject to ecological fallacy. Other studies have found a positive association between primary care use and the probability of ACSC hospitalization [18, 19], prompting concerns that ACSC hospitalizations are a marker of other factors (e.g., social determinants of health or comorbid conditions) beyond access to high quality primary care [20, 21]. Supply-side factors, including available hospital beds per capita [22] and geographic proximity to a hospital [23], have also been associated with more ACSC hospitalizations, and hospital-level admission rates vary significantly [24].

In short, evidence regarding the relationship between primary care and ACSC hospitalizations is mixed, and it remains relatively understudied among dual eligibles. Prior studies using person-level data found that receiving primary care at community health centers is associated with both increases and decreases in ACSC hospitalizations among different subgroups of dual eligibles [25–27], but these studies are limited by not accounting for other non-health center sources of primary care used by dual eligibles. Thus, the aim of this paper is to use comprehensive person-level data to examine the relationship between primary care visits and subsequent ACSC hospitalizations among the national population of fee-for-service (FFS) dual eligibles age 65 and older.

## Methods

### Data and sample

In this retrospective study, we used 2012–2018 Medicare inpatient, outpatient, and carrier for claims data on all adults age 65 and over who were dually enrolled in both FFS Medicare and Medicaid. Because Medicare is the primary payer for dual eligibles, Medicare claims are sufficient for identifying all outpatient primary care visits and hospital-based care [4]. We restricted our sample to individuals age 65 and over, without end-stage renal disease, who were dually eligible and enrolled in Medicare Parts A and B and Medicaid for all 12 months in a given calendar year, and who had at least one Medicare claim during that year. Because we used a lagged independent variable, we also required individuals to have at least two consecutive years of enrollment during the study period. For those with discontinuous enrollment, any enrollment spells of two or more years were included. We also excluded individuals with more than 365 outpatient visits during the year, as values exceeding this seemed infeasible and represented less than 0.01% of our total sample.

### Analysis

Using linear probability models (LPMs), we estimated the likelihood of dual eligibles experiencing an ACSC hospitalization during the year as a function of their primary care use in the prior year as shown in the following equation:

$$\Pr {\left( ACSC\ Hosp\right)}_{ijt}={\alpha}_0+{\beta}_1{PCP}_{it-1}+{\beta}_2{Specialty}_{it-1}+{\beta}_3{X}_{it}+{\beta}_4{County}_j+{\beta}_5{Year}_j+{\varepsilon}_{ijt}$$

where *ACSC Hosp* is a binary variable equal to 1 if individual *i* living in county *j* experienced 1 or more ACSC hospitalizations in year *t* and 0 otherwise. We defined ACSC hospitalizations using the 2019 version of the Agency for Healthcare Research and Quality’s Prevention Quality Indicators [28]. Our key independent variable (*PCP*) is a count of an individual’s outpatient evaluation and management (E&M) visits to primary care providers (PCPs) in year *t-1*. Using provider type codes, we defined primary care providers as physicians in general practice, family practice, internal medicine, or geriatrics and nurse practitioners, certified clinical nurse specialists, or physician assistants with at least one claim for evaluation and management (E&M) services. Our definition of primary care providers also included E&M visits to federally qualified health centers and rural health clinics, which bill as facilities rather than individual providers. These were identified using the last 4 digits of the Centers for Medicare and Medicaid Services Certification Number. To account for an individual’s overall outpatient care use, we also included a count of outpatient E&M visits to specialists (*Specialty*), defined as non-primary care physicians. Because individuals may use more primary care after hospitalization, we lagged both outpatient variables by 1 year to ensure that our models captured the directionality of the relationship between primary care use in 1 year and ACSC hospitalization the next year. Finally, we assessed collinearity between primary care and outpatient specialty visits and conducted sensitivity analyses excluding outpatient specialty visits.

We also adjusted for demographic characteristics (*X*) including age, sex, race/ethnicity, an indicator for disability derived from the original reason for Medicare eligibility, and a categorical measure of the number of chronic conditions reported in the Chronic Conditions Warehouse. We derived race/ethnicity data from the Research Triangle Institute enhanced race codes created by a validated algorithm of surnames and home addresses in Medicare claims data [29]. To account for variation in state-level Medicaid payment policy (e.g., Medicaid managed care and differing payment policies) [30–32] while also adjusting for unobserved county-level heterogeneity, we included county-level fixed effects (*County*). Prior studies have shown that ACSC hospitalizations generally decreased over time while observation stays increased [33, 34]. Therefore, we also included year fixed effects (*Year)* to control for such secular trends. Given repeated observations of individuals over time, we clustered standard errors at the individual level.

Because availability of and distance to PCPs and hospitals may be a factor in ACSC hospitalization, we stratified our models by rurality (urban vs. rural using urban influence codes) [35]. Stratification was necessary because otherwise our county-level rurality measure would be subsumed by the county-level fixed effects in our models. Additionally, since roughly half of ACSC hospitalization definitions require a diabetes or congestive heart failure (CHF) diagnosis, we were concerned that a different underlying prevalence of these conditions in our sample might lead to spurious correlations. Therefore, we conducted subgroup analyses on individuals with diabetes or CHF [28]. Finally, because individuals in nursing facilities have higher rates of potentially-avoidable hospitalization [36], we also conducted sensitivity analyses excluding any individuals who ever received care in a nursing facility during the year (*N* = 2,663,666 person-years). Given our large sample size, nearly all results are statistically significant. Thus, we focus on reporting the direction and magnitude of our findings. This study was deemed exempt human subjects research under Category 4 of the Common Rule (45 CFR 46) by the University of North Carolina at Chapel Hill Institutional Review Board.

## Results

### Summary statistics

Our sample included 10,461,127 person-year observations across the 2012–2018 study period. Of these, 1,707,535 (16.3%) received no primary care, 7,230,068 (69.1%) had at least one primary care visit in a calendar year, and 1,523,524 (14.6%) used primary care exclusively (Table 1). Overall, those who did not receive primary care were younger, more likely to be male and Black, and had fewer chronic conditions compared to primary care users. Non-primary care users did receive some outpatient specialty care but had fewer visits on average compared to primary care users who also used outpatient specialty care. Finally, a higher percentage of those who received some primary care had an ACSC hospitalization compared to non-primary care users.

**Table 1:** Characteristics of dual eligibles age 65 and over, by primary care use

Variable	Received no primary care	Received some primary care	Received only primary care
N (person-years)	1,707,535	7,230,068	1,523,524
Mean age	76.5^***^ (8.1)	78.2^***^ (8.6)	76.8^***^ (8.1)
% Male	38.5^***^	30.0^***^	31.3^***^
**Race/Ethnicity**			
% White	48.1^***^	55.1^***^	41.4^***^
% Black	17.5^***^	14.9^***^	15.6^***^
% Asian/Pacific Islander	10.3^***^	10.9^***^	19.6^***^
% AI/AN	2.7^***^	0.9^***^	1.0^***^
% Hispanic	20.2^***^	17.0^***^	21.2^***^
% Other	1.2^***^	1.2^***^	1.2^***^
**Disability Status**			
% Older adult with disability	23.4^***^	27.9^***^	21.4^***^
**Residence**			
% Urban	79.4^***^	83.1^***^	79.1^***^
% Rural	20.6^***^	16.9^***^	20.9^***^
**Health Status**			
Mean # of chronic conditions	3.0^***^ (2.9)	6.2^***^ (2.9)	3.5^***^ (2.3)
% with 0 chronic conditions	28.2^***^	0.9^***^	7.4^***^
% with 1–3 chronic conditions	32.6^***^	17.5^***^	46.9^***^
% with 4–6 chronic conditions	25.6^***^	38.7^***^	34.9^***^
% with 7–9 chronic conditions	10.9^***^	29.7^***^	9.2^***^
% with 10+ chronic conditions	2.7^***^	13.2^***^	1.6^***^
**Health Services Use**			
Mean PCP visits	0.0^***^ (0.0)	9.6^***^ (9.0)	5.2^***^ (4.6)
Mean outpatient specialist visits	4.5^***^ (6.6)	10.5^***^ (11.2)	0.0^***^ (0.0)
**ACSC Hospitalizations**			
Mean # of ACSC hospitalizations	0.05^***^ (0.3)	0.1^***^ (0.5)	0.04^***^ (0.3)
% with 1+ ACSC hospitalization	3.6^***^	9.0^***^	3.3^***^

Note: Sample includes individuals ages 65 and older with at least 2 consecutive observations in 2012–2018, a maximum of 365 total outpatient visits in any year, and no missing race/ethnicity or sex data. Ranksum test for continuous variables or chi-squared test on categorial variables to test differences between individuals who did not receive primary care, who did receive some primary care, and who received only primary care

^*^p < 0.05

^**^p < 0.01

^***^p < 0.001

### Regression results

The results from our LPMs are presented in Table 2. We found that in both urban and rural counties, a larger number of primary care visits was associated with a lower probability of ACSC hospitalization. Specifically, each additional primary care visit was associated with a decrease in the probability of ACSC hospitalization of 0.059 percentage points (95% CI: − 0.061 to − 0.057) in urban areas and 0.026 percentage points (95% CI: − 0.031 to − 0.021) in rural areas. However, this relationship was not unique to primary care. We also found a similar relationship between the total number of outpatient specialty visits and the probability of ACSC hospitalization. Each additional outpatient specialty visit was associated with a lower probability of ACSC hospitalization by 0.067 percentage points (95% CI: − 0.69, − 0.065) for urban residents and 0.146 percentage points (95% CI: − 0.150, − 0.142) for rural residents.

**Table 2:** Adjusted associations between PCP and specialty visits and ACSC hospitalization among urban and rural dual eligibles age 65 and over

Variable	Urban	Rural
N (person-years)	8,562,571	1,894,368
PCP visits	− 0.059^***^ (0.002)	− 0.026^***^ (0.005)
Outpatient specialist visits	−0.067^***^ (0.002)	− 0.146^***^ (0.004)
Age	0.037^***^ (0.001)	0.025^***^ (0.003)
Male	−0.138^***^ (0.021)	0.336^***^ (0.050)
**Race/Ethnicity**		
Black	1.599^***^ (0.034)	−0.827^***^ (0.088)
Asian/Pacific Islander	−0.707^***^ (0.028)	−0.787^***^ (0.192)
AI/AN	1.215^***^ (0.147)	0.864^***^ (0.173)
Hispanic	0.399^***^ (0.029)	−0.471^***^ (0.108)
Other	−0.795^***^ (0.080)	−0.539 (0.333)
**Chronic Conditions**		
1–3 conditions	1.129^***^ (0.011)	1.606^***^ (0.030)
4–6 conditions	4.149^***^ (0.017)	5.815^***^ (0.044)
7–9 conditions	12.130^***^ (0.031)	16.440^***^ (0.074)
10+ conditions	29.970^***^ (0.060)	36.280^***^ (0.136)
**Disability Status**		
With Disability	1.106^***^ (0.026)	0.371^***^ (0.053)
**Year**		
2014	−0.339^***^ (0.027)	−0.566^***^ (0.063)
2015	−0.646^***^ (0.028)	−1.242^***^ (0.065)
2016	−0.508^***^ (0.028)	−1.292^***^ (0.066)
2017	−0.689^***^ (0.028)	−1.459^***^ (0.066)
2018	−0.949^***^ (0.028)	−1.830^***^ (0.067)
Constant	−2.564^***^ (0.101)	−0.739^***^ (0.230)

Note: Reference categories are female, non-Hispanic White, 0 chronic conditions, and year 2013. Coefficients are interpreted as percentage point changes with probability of outcome scaled from 0 to 100. For example, the coefficient of − 0.059 for PCP visits among urban dual eligibles means that each PCP visit is associated with a decrease in risk of ACSC hospitalization by 0.059 percentage points

^**^p < 0.05

^***^p < 0.01, Robust standard errors in parentheses

We further stratified our main models by age of individuals: (1) ages 65–89 and (2) ages 90 and over (Additional file 1: Appendix Table 1). In general, the relationship between primary visits and ACSC hospitalizations remained similar in both groups regardless of rurality. However, duals ages 90+ had a larger predicted magnitude of decreased risk for ACSC hospitalization in both urban and rural groups compared to duals ages 65–89.

We found a moderate correlation (0.58) between primary care and outpatient specialty visits. In our sensitivity analyses excluding outpatient specialty visits, we observed increases up to 0.075 percentage points in the magnitude of the primary care visit coefficients, though the direction of the association remained the same (Additional file 1: Appendix Table 2).

Other factors were also associated with the probability of ACSC hospitalization, and this often varied across urban and rural counties. For example, in rural counties the probability of ACSC hospitalization was lower among dual eligibles who are Black, Asian, or Hispanic compared to Whites, while in urban counties the relationship was more mixed. Similarly, male sex was associated with a lower risk of ACSC hospitalization by 0.138 percentage points hospitalization in urban counties, but a higher risk of 0.336 percentage points in rural counties. Other factors related to health status—like age and number of chronic conditions—were consistently associated with an higher probability of ACSC hospitalization across urban and rural counties. Our findings remained consistent when excluding individuals who received any care in a nursing facility during the year (Table 3).

**Table 3:** Adjusted associations between PCP and specialty visits and ACSC hospitalization among urban and rural dual eligibles age 65 and over, excluding nursing facility users

Variable	Urban	Rural
N (person-years)	6,438,381	1,354,892
PCP visits	−0.056^***^ (0.002)	− 0.002 (0.005)
Outpatient specialist visits	−0.059^***^ (0.002)	− 0.089^***^ (0.006)
Age	0.066^***^ (0.001)	0.067^***^ (0.003)
Male	0.011 (0.012)	0.596^***^ (0.051)
**Race/Ethnicity**		
Black	1.408^***^ (0.035)	−1.039^***^ (0.094)
Asian/Pacific Islander	−0.522^***^ (0.027)	−0.762^***^ (0.183)
AI/AN	1.187^***^ (0.140)	0.934^***^ (0.171)
Hispanic	0.473^***^ (0.028)	−0.635^***^ (0.107)
Other	−0.815^***^ (0.075)	−0.708^**^ (0.330)
**Chronic Conditions**		
1–3 conditions	1.019^***^ (0.012)	1.277^***^ (0.031)
4–6 conditions	3.715^***^ (0.019)	5.024^***^ (0.050)
7–9 conditions	10.990^***^ (0.037)	15.320^***^ (0.093)
10+ conditions	26.010^***^ (0.083)	34.320^***^ (0.198)
**Disability Status**		
With Disability	0.930^***^ (0.026)	0.227^***^ (0.055)
**Year**		
2014	−0.281^***^ (0.026)	− 0.493^***^ (0.067)
2015	−0.495^***^ (0.027)	−1.042^***^ (0.068)
2016	−0.469^***^ (0.027)	−1.077^***^ (0.069)
2017	−0.671^***^ (0.027)	−1.247^***^ (0.070)
2018	−0.890^***^ (0.028)	−1.608^***^ (0.071)
Constant	−5.049^***^ (0.107)	−4.162^***^ (0.262)

Note: Reference categories are female, non-Hispanic White, 0 chronic conditions, and year 2013. Coefficients are interpreted as percentage point changes with probability of outcome scaled from 0 to 100. For example, the coefficient of − 0.056 for PCP visits among urban dual eligibles means that each PCP visit is associated a lower risk of ACSC hospitalization by 0.056 percentage points

^**^p < 0.05

^***^p < 0.01, Robust standard errors in parentheses

In our diabetes and CHF subgroup analyses, we found not only that primary care visits continued to be associated with a lower probability of ACSC hospitalization, but also that the strength of the association increased (Table 4). For those with diabetes, each additional primary care visit was associated with a decrease in the probability of ACSC hospitalization of 0.061 percentage points in urban counties (95% CI: − 0.064 to − 0.058) and 0.034 percentage points in rural counties (95% CI: − 0.041 to − 0.027). For those with CHF, each additional primary care visit was associated with a decrease in the probability of ACSC hospitalization of 0.112 percentage points in urban counties (95% CI: − 0.116 to − 0.108) and 0.046 percentage points in rural counties (95% CI: − 0.047 to − 0.045). As in our main models, each outpatient specialist visit was also associated with a lower probability of ACSC hospitalization.

**Table 4:** Adjusted Associations between PCP and specialty visits and ACSC hospitalization among urban and rural dual eligibles age 65 and over with diabetes or CHF

Variable	Diabetes		CHF	
	Urban	Rural	Urban	Rural
N (person-years)	3,819,765	782,184	2,131,443	530,800
PCP visits	−0.061^***^ (0.003)	− 0.034^***^ (0.007)	−0.112^***^ (0.004)	− 0.0462^***^ (0.001)
Outpatient specialist visits	−0.061^***^ (0.002)	− 0.137^***^ (0.007)	−0.122^***^ (0.003)	− 0.275^***^ (0.009)
Age	0.035^***^ (0.002)	0.008 (0.005)	−0.049^***^ (0.004)	−0.073^***^ (0.007)
Male	−0.360^***^ (0.036)	0.297^***^ (0.089)	−0.888^***^ (0.063)	0.022 (0.138)
**Race/Ethnicity**				
Black	2.496^***^ (0.057)	−0.436^***^ (0.148)	3.487^***^ (0.092)	−0.583^**^ (0.227)
Asian/Pacific Islander	−0.335^***^ (0.050)	−0.942^***^ (0.350)	0.696^***^ (0.111)	−0.185 (0.886)
AI/AN	1.700^***^ (0.248)	1.364^***^ (0.291)	2.883^***^ (0.473)	2.685^***^ (0.512)
Hispanic	1.155^***^ (0.051)	−0.079 (0.192)	2.407^***^ (0.098)	0.813^**^ (0.375)
Other	−0.632^***^ (0.128)	−0.307 (0.608)	− 0.464^*^ (0.237)	−1.115 (1.062)
**Chronic Conditions**				
2–3 conditions	0.685^***^ (0.022)	1.182^***^ (0.069)	2.102^***^ (0.125)	2.463^***^ (0.359)
4–6 conditions	3.311^***^ (0.027)	4.894^***^ (0.080)	6.982^***^ (0.121)	8.516^***^ (0.350)
7–9 conditions	11.490^***^ (0.042)	15.990^***^ (0.112)	16.860^***^ (0.125)	20.310^***^ (0.356)
10+ conditions	30.280^***^ (0.073)	37.270^***^ (0.173)	35.170^***^ (0.136)	39.550^***^ (0.376)
**Disability Status**				
With Disability	1.136^***^ (0.044)	0.171 (0.091)	1.187^***^ (0.076)	−0.081 (0.143)
**Year**				
2014	−0.514^***^ (0.045)	− 0.713^***^ (0.112)	−0.688^***^ (0.078)	− 0.801^***^ (0.169)
2015	−1.227^***^ (0.048)	−1.664^***^ (0.115)	−1.539^***^ (0.082)	−2.283^***^ -(0.175)
2016	−1.294^***^ (0.048)	−2.035^***^ (0.117)	−1.415^***^ (0.083)	− 2.255^***^ (0.178)
2017	−1.346^***^ (0.048)	−2.288^***^ (0.118)	− 1.200^***^ (0.085)	− 2.490^***^ (0.181)
2018	− 1.648^***^ (0.049)	− 2.738^***^ (0.121)	− 1.661^***^ (0.085)	−3.272^***^ (0.184)
Constant	− 2.499^***^ (0.181)	0.702 (0.431)	4.181^***^ (0.317)	9.079^***^ (0.709)

Note: CHF = Congestive Heart Failure. Reference categories are female, non-Hispanic White, 1 chronic condition, and year 2013. Coefficients are interpreted as percentage point changes with probability of outcome scaled from 0 to 100. For example, the coefficient of − 0.061 for PCP visits among urban dual eligibles with diabetes means that each PCP visit is associated with a lower probability of ACSC hospitalization by 0.061 percentage points

^**^p < 0.05

^***^p < 0.01, Robust standard errors in parentheses

## Discussion

In this study, we found that each additional primary care visit a dual eligible age 65 and over received was associated with a small absolute lower probability of ACSC hospitalization the following year. Given the 3.5% baseline rate of ACSC hospitalization in our sample, this translates to a relative risk reduction between 1.4 and 4.3% across all models. Moreover, the direction of this relationship was consistent across all models. This aligns with past studies demonstrating similar reductions in ACSC hospitalizations with greater access to and use of primary care [13–17, 37–40].

However, the magnitude of the association is more nuanced and depends on individual and geographic characteristics. The association between primary care visits and ACSC hospitalization is stronger for dual eligibles living in rural versus urban counties, and for those with diabetes and CHF compared with the entire dual eligible population. Underlying factors proxied by race and ethnicity in our analyses are also associated with both increases and decreases in the probability of ACSC hospitalization in different contexts.

While some of our findings—like the strong positive relationship between number of chronic conditions and probability of ACSC hospitalization are straightforward and as expected—other findings require speculative interpretation. First, individuals living in rural areas or who belong to certain racial or ethnic groups may be healthier in ways we cannot observe in claims data but explain their lower probability of ACSC hospitalization. Alternatively, the reduced probability of ACSC hospitalization may indicate barriers to hospital care stemming from rurality, access to health care resources, socioeconomic status, and racism. For example, evidence suggests urban residents with low socioeconomic status prefer to seek care at emergency departments, because after-hours care, ambulance transport, and the perception of “one-stop shopping” are more attractive than primary care [41]. The use of EDs for ACSC (vs. non-ACSC) care is more likely to result in an ACSC hospitalization [42, 43]. As such, individuals who seek care in EDs may be more prone to be admitted when their care is for an ACSC.

Furthermore, primary care is defined more by its characteristics than by the clinicians who deliver it [8]. Compared to Medicare only beneficiaries, dual eligibles have a higher physical and mental disease burden and more social needs resulting from relatively lower income and educational achievement [44]. Thus, dual eligibles may face more barriers accessing high-quality care, including difficulty finding providers accepting Medicare and Medicaid, coordinating their care, and managing medication [45, 46]. Age and racial/ethnic discrimination may compound these difficulties [45]. Given these barriers, dual eligibles may require more help—beyond the PCP—to navigate the health care system [47]. Dual eligibles also struggle with patient-provider communication, which presents further challenges [48].

Primary care visits were not unique in their association with a lower probability of ACSC hospitalization. Each outpatient specialist visit a dual eligible receives is also associated with a similar—and sometimes even greater—decrease in the probability of ACSC hospitalization. This suggests that any evaluation and management in outpatient settings may be beneficial, regardless of who provides it. This important because individuals who never used primary care during the year did visit outpatient specialists roughly as often as individuals who exclusively used primary care visited their PCPs. Thus, this population may have health conditions that require regular visits with specialists, and they may rely on their close relationship with those specialists to serve as their PCPs.

Our study has several limitations. First, the analysis excludes dual eligibles enrolled in Medicare managed care and younger dual eligibles with disabilities. Therefore, our findings may not be generalizable to these populations. Second, we define primary care as E&M services delivered by clinicians in general practice, family practice, internal medicine, or geriatrics. However, some dual eligibles may have received primary care from other specialties (e.g., obstetrics/gynecology, cardiology, pulmonology). Our reliance on claims data precludes us from identifying instances when such specialists are serving as a patient’s PCP. To the extent that this occurs, however, it will bias our primary results towards the null. Third, there may be unobserved differences between primary care users and nonusers for which we cannot account using claims data. Finally, our study does not assess primary care continuity. If the benefit of primary care lies in the ongoing patient-provider relationship, our results may again be biased towards the null by considering all primary care to be equivalent regardless of continuity.

## Conclusions

Overall, our findings suggest that while increased outpatient care—and primary care in particular—may reduce ACSC hospitalizations among dual eligibles age 65 and older, the relationship is complex. Dual eligibles have extensive health needs, face substantial barriers to care, and likely need additional support to navigate the health care system and establish more coordinated, continuous care. Future work should focus on identifying and meeting these needs because efforts to increase primary care use among dual eligibles most likely need to be tailored based on clinical, sociodemographic, geographic, and other factors.

## Supplementary Information

**Additional file 1: Appendix Table 1.** Adjusted Associations between PCP and Specialty Visits and ACSC Hospitalization among Urban and Rural Dual Eligibles Age 65 and Over, Stratified by Age. **Appendix Table 2.** Adjusted Associations between PCP and Specialty Visits and ACSC Hospitalization among Urban and Rural in Dual Eligibles Age 65 and Over, Excluding Specialty Visits

## Data Availability

The datasets analyzed in this study include protected health information and are not publicly available due to privacy concerns. Researchers interested in obtaining and using the data must request them from the Centers for Medicare and Medicaid Services via a formal process guided by the Research Data Assistance Center (ResDAC).

## Abbreviations

ACSC: Ambulatory care sensitive condition(s)
FFS: Fee-for-service
PCP: Primary care provider
LPM: Linear probability model(s)
CHF: Congestive heart tailure
