# Supplementary material for: The association between primary care use and potentially-preventable hospitalization among dual eligibles age 65 and over
Source: BMC Health Serv Res. 2022 Jul 19;22:927. doi: 10.1186/s12913-022-08326-2 (PMC9295296; doi:10.1186/s12913-022-08326-2)
Supplement: Supplementary file 1 — Additional file 1: Appendix Table 1. Adjusted Associations between PCP and Specialty Visits and ACSC Hospitalization among Urban and Rural Dual Eligibles Age 65 and Over, Stratified by Age. Appendix Table 2. Adjusted Associations between PCP and Specialty Visits and ACSC Hospitalization among Urban and Rural in Dual Eligibles Age 65 and Over, Excluding Specialty Visits [file 12913_2022_8326_MOESM1_ESM.docx]

| **Appendix Table 1. Adjusted Associations between PCP and Specialty Visits and ACSC Hospitalization among Urban and Rural Dual Eligibles Age 65 and Over, Stratified by Age** | | | | |
| --- | --- | --- | --- | --- |
|  | **Ages 65-89** | | **Ages 90 and over** | |
| Variable | Urban | Rural | Urban | Rural |
| N (person-years) | 7,645,834 | 1,682,002 | 916,735 | 212,366 |
| PCP visits | -0.049*** | -0.076*** | -0.108*** | -0.138*** |
|  | (0.0009) | (0.003) | (0.002) | (0.006) |
| Outpatient specialist visits | -0.086*** | -0.126*** | -0.025*** | -0.016 |
|  | (0.002) | (0.007) | (0.007) | (0.024) |
| Age | 0.017*** | 0.009** | 0.075*** | 0.057*** |
|  | (0.002) | (0.004) | (0.009) | (0.020) |
| Male^a^ | -0.131*** | 0.319*** | -0.751*** | 0.187 |
|  | (0.021) | (0.051) | (0.087) | (0.216) |
| **Race/Ethnicity^b^** |  |  |  |  |
| Black | 1.454*** | -0.973*** | 2.436*** | 0.496 |
|  | (0.036) | (0.093) | (0.109) | (0.276) |
| Asian/Pacific Islander | -0.916*** | -1.020*** | 1.177*** | 1.983** |
|  | (0.029) | (0.195) | (0.113) | (0.808) |
| AI/AN | 0.991*** | 0.612*** | 3.535*** | 2.286*** |
|  | (0.150) | (0.176) | (0.651) | (0.845) |
| Hispanic | 0.185*** | -0.529*** | 2.644*** | 0.850 |
|  | (0.030) | (0.111) | (0.117) | (0.469) |
| Other | -0.964*** | -0.430 | 1.197*** | -2.268 |
|  | (0.081) | (0.339) | (0.326) | (1.450) |
| **Chronic Conditions^c^** |  |  |  |  |
| 1-3 conditions | 1.117*** | 1.634*** | 2.134*** | 2.801*** |
|  | (0.011) | (0.030) | (0.048) | (0.176) |
| 4-6 conditions | 4.095*** | 5.787*** | 6.040*** | 7.716*** |
|  | (0.018) | (0.047) | (0.062) | (0.196) |
| 7-9 conditions | 12.040*** | 16.450*** | 14.300*** | 17.960*** |
|  | (0.033) | (0.081) | (0.090) | (0.245) |
| 10+ conditions | 29.920*** | 36.480*** | 31.590*** | 36.070*** |
|  | (0.065) | (0.146) | (0.160) | (0.405) |
| **Disability Status** |  |  |  |  |
| With Disability | 0.983*** | 0.284*** | 0.855*** | 1.110*** |
|  | (0.027) | (0.054) | (0.153) | (0.301) |
| **Year** |  |  |  |  |
| 2014 | -0.290*** | -0.501*** | -0.787*** | -1.133*** |
|  | (0.028) | (0.066) | (0.092) | (0.202) |
| 2015 | -0.631*** | -1.212*** | -0.870*** | -1.571*** |
|  | (0.029) | (0.068) | (0.097) | (0.208) |
| 2016 | -0.510*** | -1.231*** | -0.678*** | -1.786*** |
|  | (0.029) | (0.069) | (0.097) | (0.211) |
| 2017 | -0.710*** | -1.356*** | -0.808*** | -2.084*** |
|  | (0.029) | (0.070) | (0.098) | (0.215) |
| 2018 | -0.962*** | -1.746*** | -1.244*** | -2.189*** |
|  | (0.029) | (0.071) | (0.099) | (0.219) |
| Constant | -1.005*** | 0.468 | -7.304*** | -4.500** |
|  | (0.125) | (0.290) | (0.887) | (1.898) |
| ** p<0.05, *** p<0.01, Robust standard errors in parentheses | | | | |

Note: Reference categories are female, non-Hispanic White, 0 chronic conditions, and year 2013. Coefficients are interpreted as percentage point changes with probability of outcome scaled from 0 – 100. For example, the coefficient of -0.049 for PCP visits among urban dual eligibles means that each PCP visit is associated with a decrease in risk of ACSC hospitalization by 0.049 percentage points.

**Appendix Table 2. Adjusted Associations between PCP and Specialty Visits and ACSC Hospitalization among Urban and Rural in Dual Eligibles Age 65 and Over, Excluding Specialty Visits**

| Variable | Main Model | | | Excluding Nursing Facility Users | | | | |  |
| --- | --- | --- | --- | --- | --- | --- | --- | --- | --- |
|  | Urban | Rural | Urban | | Rural | | | | |
| N (person-years) | 8,562,546 | 1,894,367 | 6,438,356 | | 1,354,890 | | | | |
| PCP visits | -0.107*** | -0.101*** | -0.063*** | | -0.008 | | | | |
|  | (0.002) | (0.004) | (0.002) | | (0.005) | | | | |
| Age | 0.035*** | 0.020*** | 0.069*** | | 0.070*** | | | | |
|  | (0.001) | (0.003) | (0.001) | | (0.003) | | | | |
| Male | -0.153*** | 0.318*** | 0.011 | | 0.614*** | | | | |
|  | (0.021) | (0.050) | (0.020) | | (0.051) | | | | |
| **Race/Ethnicity** |  |  |  | |  | | | | |
| Black | 1.635*** | -0.700*** | 1.442*** | | -1.017*** | | | | |
|  | (0.034) | (0.088) | (0.035) | | (0.094) | | | | |
| Asian/Pacific Islander | -0.507*** | -0.641*** | -0.427*** | | -0.697*** | | | | |
|  | (0.028) | (0.192) | (0.028) | | (0.183) | |  |  |  |
| AI/AN | 1.154*** | 0.635*** | 1.125*** | | 0.777*** | | |  |  |
|  | (0.147) | (0.173) | (0.144) | | (0.171) | | |  |  |
| Hispanic | 0.505*** | -0.309*** | 0.528*** | | -0.601*** | | |  |  |
|  | (0.029) | (0.108) | (0.028) | | (0.107) | | |  |  |
| Other | -0.701*** | -0.498 | -0.778*** | | -0.692** | | |  |  |
|  | (0.080) | (0.333) | (0.075) | | (0.331) | | |  |  |
| **Chronic Conditions** |  |  |  | |  | | |  |  |
| 1-3 conditions | 1.091*** | 1.492*** | 0.905*** | | 1.134*** | | |  |  |
|  | (0.011) | (0.029) | (0.011) | | (0.029) | | |  |  |
| 4-6 conditions | 4.021*** | 5.518*** | 3.487*** | | 4.742*** | | |  |  |
|  | (0.017) | (0.043) | (0.018) | | (0.046) | | |  |  |
| 7-9 conditions | 11.880*** | 15.920*** | 10.610*** | | 14.860*** | | |  |  |
|  | (0.030) | (0.072) | (0.034) | | (0.088) | | |  |  |
| 10+ conditions | 29.560*** | 35.560*** | 25.420*** | | 33.700*** | | |  |  |
|  | (0.059) | (0.134) | (0.081) | | (0.195) | | |  |  |
| **Disability Status** | 1.040*** | 0.306*** | 0.879*** | | 0.193*** | | |  |  |
| With Disability | (0.026) | (0.053) | (0.026) | | (0.055) | | |  |  |
|  |  |  |  | |  | | |  |  |
| **Year** | -0.366*** | -0.597*** | -0.299*** | | -0.505*** | | | | |
| 2014 | (0.027) | (0.063) | (0.026) | | (0.067) | | | | |
|  | -0.674*** | -1.274*** | -0.515*** | | -1.053*** | | | | |
| 2015 | (0.028) | (0.065) | (0.027) | | (0.068) | | | | |
|  | -0.524*** | -1.316*** | -0.488*** | | -1.095*** | | | | |
| 2016 | (0.028) | (0.066) | (0.027) | | (0.069) | | | | |
|  | -0.689*** | -1.437*** | -0.680*** | | -1.258*** | | | | |
| 2017 | (0.028) | (0.066) | (0.027) | | (0.067) | | | | |
|  | -0.943*** | -1.790*** | -0.894*** | | -1.617*** | | | | |
| 2018 | (0.028) | (0.067) | (0.028) | | (0.071) | | | | |
|  |  |  |  | |  | | | | |
| Constant | -2.466*** | -0.390* | -5.289*** | | -4.477*** | | |  |  |
|  | (0.101) | (0.230) | (0.107) | | (0.262) | | |  |  |
| ** p<0.05, *** p<0.01, Robust standard errors in parentheses | | | |  | |  | | |  |

Reference categories are female, non-Hispanic White, 1 chronic condition, and year 2013. Coefficients are interpreted as percentage point changes with probability of outcome scaled from 0 – 100. For example, the coefficient of -0.107 for PCP visits among urban dual eligibles in our main model means that each PCP visit is associated with an 0.107 percentage point reduction in the probability of having an ACSC hospitalization.
